# Hyperhomocysteinemia and Accelerated Aging: The Pathogenic Role of Increased Homocysteine in Atherosclerosis, Osteoporosis, and Neurodegeneration

**DOI:** 10.7759/cureus.42259

**Published:** 2023-07-21

**Authors:** Hussam Alkaissi, Samy I. McFarlane

**Affiliations:** 1 Internal Medicine, Kings County Hospital Center, Brooklyn, USA; 2 Internal Medicine, Veterans Affairs Medical Center, Brooklyn, USA; 3 Internal Medicine, State University of New York Downstate Medical Center, Brooklyn, USA; 4 Endocrinology, State University of New York Downstate Medical Center, Brooklyn, USA

**Keywords:** accelerated aging, homocysteine metabolism, hyperhomocysteinemia, classical homocystinuria, homocystinuria, pathogenesis, thrombophilia, cardiovascular disease, atherosclerosis, osteoporosis

## Abstract

Cardiovascular diseases and osteoporosis, seemingly unrelated disorders that occur with advanced age, share major pathogenetic mechanisms contributing to accelerated atherosclerosis and bone loss. Hyperhomocysteinemia (hHcy) is among these mechanisms that can cause both vascular and bone disease. In its more severe form, hHcy can present early in life as homocystinuria, an inborn error of metabolic pathways of the sulfur-containing amino acid methionine. In its milder forms, hHcy may go undiagnosed and untreated into adulthood. As such, hHcy may serve as a potential therapeutic target for cardiovascular disease, osteoporosis, thrombophilia, and neurodegeneration, collectively representing accelerated aging. Multiple trials to lower cardiovascular risk and improve bone density with homocysteine-lowering agents, yet none has proven to be clinically meaningful. To understand this unmet clinical need, this review will provide mechanistic insight into the pathogenesis of vascular and bone disease in hHcy, using homocystinuria as a model for accelerated atherosclerosis and bone density loss, a model for accelerated aging.

## Introduction and background

Cardiovascular disease (CVD) and osteoporosis, seemingly unrelated disorders that occur with advanced age, share major pathogenetic mechanisms contributing to atherosclerosis and osteoporosis. Previous work by our group highlighted the relationship between these two common disorders [1-3]. Hyperhomocysteinemia (hHcy) is one of the etiologies underlying accelerated atherosclerosis and osteoporosis, which goes undiagnosed and untreated in milder forms and might serve as a potential therapeutic target for CVD, osteoporosis, and thrombophilia. In this review, we discuss the history and the discovery of homocystinuria (HCU) in 1962 and its emerging role as a common underlying factor in accelerated CVD, thrombophilia, and bone disease. We also discuss the epidemiology of HCU and screening methods and the epidemiology of hHcy related to CVD and bone disease. We provide a detailed and illustrated pathophysiology of HCU, including genetics, biochemistry, and mechanisms of end-organ damage. Finally, we discuss the cutting-edge interventions regarding the diagnosis and treatment of this disorder and provide a section on future directions and potential research and clinical trial focus, including enzyme replacement therapy as a promising treatment option for HCU, preclinical and early clinical trials of cystathionine-ß-synthase (CBS) and cystathionine-γ-lyase (CGL) replacement therapies that are underway.

## Review

History

In 1962, in an attempt to identify biochemical etiologies of developmental delay, Carson and Neill examined over 2000 affected children in Ireland using chemical testing and chromatography. They were able to identify two young females with seizures, a low IQ of 30, and very high urine levels of sulfur-containing amino acid, which was later found to be homocysteine (Hcy) [4]. Two years later, biological samples were sent to the National Institutes of Health (NIH), where the biochemical defect was determined to be a low activity of the CBS enzyme by Mudd and others [5].

Epidemiology of HCU

Incidence of classical HCU varies from 1:1,800 to 1:1,000,000 births, classifying HCU as a rare inborn error of metabolism, at least in the Western hemisphere, where consanguinity rates are low. The highest prevalence is found in Qatar [6]. Milder cases of hHcy are much more common, such as methyltetrahydrofolate reductase gene (*MTHFR*) C677T polymorphism. Its prevalence ranges from 1-2% in African-Americans, 10-14% in Caucasians, and up to 25% in Hispanic individuals. A study in Japan showed enrichment of *MTHFR* polymorphism in younger age groups as opposed to older ones, with 19% prevalence in the age group of 14-55 years, down to 7% prevalence in individuals above 80 years of age [7]. Such a drop in prevalence with aging points toward non-carrier survival and higher mortality in individuals with *MTHFR* polymorphism, which was established in later studies that showed higher mortality with *MTHFR* polymorphism, especially in men [8]. If *MTHFR* polymorphism is deleterious, then why is it highly prevalent? Such an increase in prevalence is not fully explained, especially considering that *MTHFR* polymorphism is associated with fetal loss. One explanation is that widespread folate supplementation during pregnancy might have relieved the selective pressure against the CT and TT genotype, resulting in their subsequent increase in the gene pool [9,10] 

Newborn Screening

Since methionine levels are almost always elevated in HCU, it is used as a screening method for newborns. It is an indirect screening method, as Hcy is unsuitable for small samples such as the "heel prick" screening method. As such, some groups of patients are missed by the methionine screening method, especially the B6-responsive HCU phenotype [6].

Epidemiology of hHcy in CVDs

A meta-analysis of over 2,000 patients showed that folic acid supplementation effectively reduced carotid intima-media thickness (CIMT), particularly in subgroups of patients with high cardiovascular risk factors and chronic kidney disease (CKD). These patients experienced a reduction in CIMT by 0.04 mm and 0.16 mm, respectively. However, there was no significant CIMT reduction in otherwise healthy individuals with isolated mild elevation in Hcy levels. CIMT reduction was seen in patients with higher baseline CIMT of ≥ 0.8 mm with a reduction in Hcy levels of more than 30% [11]. Another meta-analysis of 14 studies encompassing over 39,000 patients showed that Hcy lowering on stroke risk was modest and only observed in regions without folate fortification in food with an average risk reduction of about 12% [12]. Another meta-analysis of 30 trials pooling over 82,000 patients showed that folic acid has a modest risk reduction of stroke and combined cardiovascular events by 10% and 4%, respectively, particularly in patients with lower folic acid levels [13]. CVD risk has been associated with S-adenosylhomocysteine (SAH), the precursor of Hcy. There is evidence that supplementation with vitamin B12 and folate targeting Hcy-lowering may fail to reduce circulating and intracellular SAH levels. Thus, such trials fail to show any clinical benefit [14]. Alternative explanations for why some trials have failed to show clinical benefit include the presence of an "endothelial memory" in the form of epigenetic changes or the trials being too short to show a meaningful benefit.

Genetics

Genetic causes of hHcy can be divided into defects in the transsulfuration pathway or defects in the remethylation pathway. Classical HCU is caused by a mutation in the *CBS* gene resulting in a defect in the transsulfuration pathway. The human *CBS* gene lies on chromosome 21, consisting of 23 exons. CBS enzyme is 551 amino acids long, with a molecular weight of 67 kDa. Pyridoxine and heme ring are added, which may help stabilize the enzyme [15,16]. Some mutations render the CBS less stable at the physiological carbon monoxide (CO) level due to the heme affinity to CO, such as mutation p.P49L. This mutation is rescued with B6 treatment [17]. CBS can be cleaved by trypsin and tumor necrosis factor-alpha (TNF-α) into a more active 50 kDa version [18]. It functions as a cytoplasmic enzyme and can be secreted from the liver and endothelial cells as plasma protein. CBS condenses Hcy with serine, producing cystathionine and water. CBS is not selective for serine and instead can use cysteine, producing the gasotransmitter, hydrogen sulfide (H_2_S) [19]. Over 150 mutations have been identified in the *CBS* gene; c.T833C (p.I287T) accounts for around 21%. While homozygous mutation is rare, heterozygous loss of function is much more common [6].

Other causes of hHcy may result in defects in the remethylation pathway that converts Hcy back to methionine. Of these, the most common is the C677T polymorphism in the *MTHFR* gene. C677T polymorphism leads to hHcy with increased cardiovascular risk and low bone mineral density but without neurological deficit [20]. Less common mutations in the remethylation pathway may occur in genes related to vitamin B12 metabolism (CblA through H) [21,22].

Biochemical abnormalities

Metabolic Pathways of Sulfur-Containing Amino Acids

**Figure 1:**
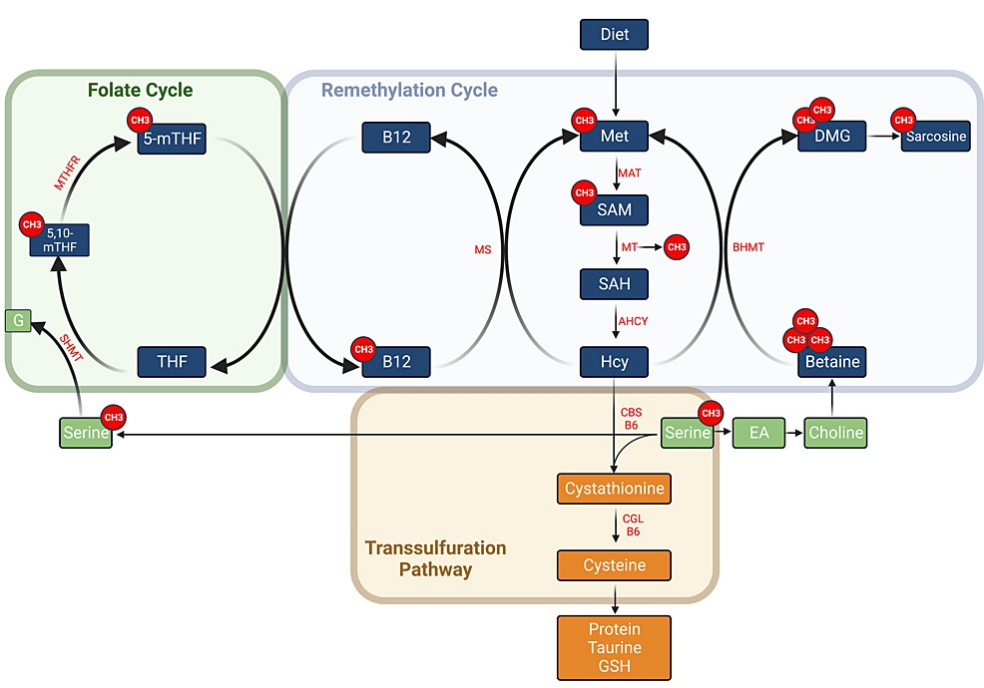
The metabolism of sulfur-containing amino acids starts with the essential amino acids. 5-mTHF: 5-methyl tetrahydrofolate; 5,10-mTHF: 5,10 methylene tetrahydrofolate; AHCY: adenosylhomocysteinase; B6: vitamin B6 (pyridoxal phosphate); B12: vitamin B12 (cyanocobalamin); BHMT: betaine homocysteine S-methyltransferase; CBS: cystathionine ß synthase; CGL: cystathionine γ-lyase; CH3: methyl group; DMG: dimethylglycine; EA: ethanolamine; G: glycine; GSH: glutathione (reduced form); Hcy: homocysteine; MAT: methionine adenosyltransferase; Met: methionine; MT: methyl transferases; MS: methionine synthase; MTHFR: methylenetetrahydrofolate reductase; SAH: S-adenosylhomocysteine; SAM: S-adenosylmethionine;  SHMT: serine hydroxymethyl transferase; THF: tetrahydrofolate Image credit: Alkaissi H and McFarlane SI; Created with BioRender.com

Humans lose the ability to synthesize methionine and acquire the essential, sulfur-containing amino acid from dietary sources. Methionine metabolism yields the synthesis of the other three sulfur-containing amino acids, namely Hcy, cysteine, and taurine (Figure 1). One of the main functions of the methionine metabolic pathway is the generation of the universal methyl donor, S-adenosylmethionine (SAM), a molecule with a central role in methylation and one-carbon metabolism. SAM is the methyl donor to DNA, RNA, proteins (lysine and arginine residues), creatine and adrenaline biosynthetic pathways, and several others. Once SAM donates the methyl group, it is converted to SAH and further to Hcy. SAH is a natural competitive inhibitor of SAM-dependent methyltransferases due to the higher affinity of binding of SAH to the enzyme than SAM; thus, the SAM/SAH ratio plays an important role in methylation and overall one-carbon metabolism. Hcy is either remethylated back to methionine by the enzyme methionine synthase (Figure 1), a vitamin B12-dependent enzyme that transfers a methyl group from N5-methyltetrahydrofolate (N5-MTHF) to convert Hcy back to methionine. Another remethylating enzyme utilizes trimethylglycine, known as betaine, catalyzed by the enzyme betaine-homocysteine S-methyltransferase (BHMT). Betaine is further demethylated to dimethylglycine (DMG) and sarcosine. As such, sarcosine can be used as an indicator of betaine intake. 

This part of the methionine pathway is referred to as the remethylation pathway, as opposed to the fate of Hcy, the transsulfuration pathway. In the transsulfuration pathway, Hcy is condensed with serine by the pyridoxine-dependent enzyme CBS. Since CBS uses pyridoxine (vitamin B6) as a coenzyme, it is used as an initial treatment for HCU patients, although only 50% of patients are responsive to B6 [6]. Cystathionine is further converted to cysteine by CGL. Cysteine can be converted to the other sulfur-containing amino acid, taurine, enter protein synthesis, or the antioxidant tripeptide glutathione [23,24].

Serine plays three important roles in Hcy metabolism. Firstly, it remethylates THF to N5, N10-methyleneTHF (that is further converted to N5-MTHF by methyltetrahydrofolate reductase MTHFR), and by doing that, serine maintains the pool of methylated THF that can recycle Hcy back to methionine. Secondly, serine can be converted to ethanolamine, then choline, then betaine, another methylation source that can recycle Hcy back to methionine through the BHMT enzyme. As mentioned above, serine condenses with Hcy to form cystathionine in the first step of the transsulfuration pathway [25,26]. 

Epigenetic, Epitranscriptomic, and Proteomic Changes in HCU 

**Figure 2:**
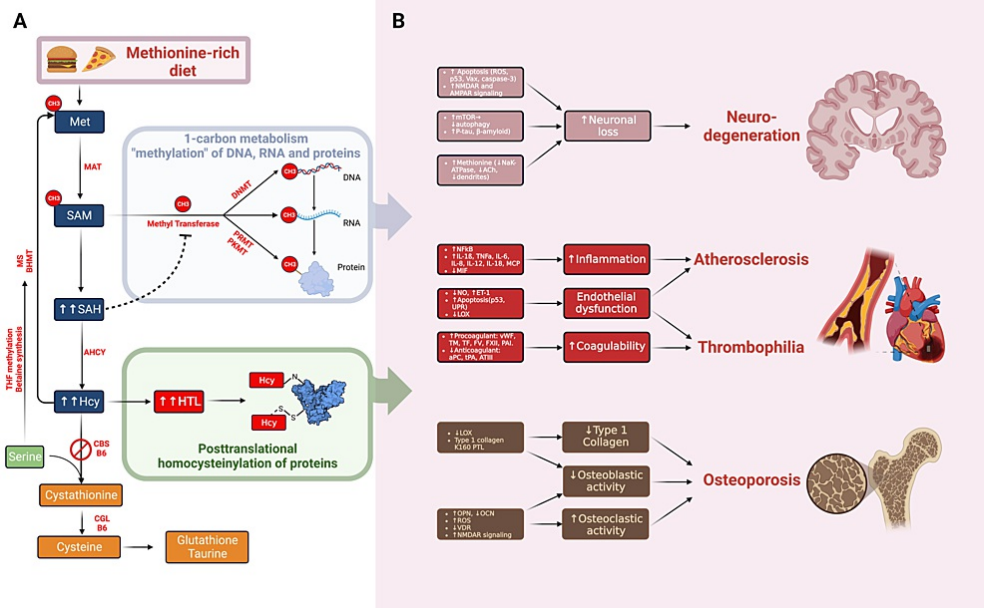
(A) The pathophysiology of HCU (and, to an extent, hyperhomocysteinemia of other causes) results from two main processes: an altered and dysregulated methylation process of DNA, RNA, and proteins. The second pathogenic process results from protein modification by Hcy and its derived product, HTL, resulting in the post-translational modification of countless proteins. Both N- and S-homocysteinylation of protein are shown. (B) These two processes (altered methylation and post-translational protein modification) can affect virtually every tissue. However, the most salient and clinically relevant features are shown: the brain, cardiovascular system, and bones. The neurodegenerative net effect of HCU results from increased neuronal apoptosis, accumulation of amyloid plaques, and reduced dendrites. The increase in thrombophilia and atherosclerotic CVD is a result of increased inflammatory cytokines, endothelial dysfunction, and a hypercoagulable state. Finally, the skeletal manifestations of HCU (predominantly loss of bone mineral density) is another complex phenomenon resulting from increased osteoclastic activity, reduced osteoblastic activity, and a reduction in collagen cross-linking. LOX is an essential enzyme in collagen cross-linking and is remarkably prone to inhibition by Hcy. This has several implications, including reduced osteoblastic activity and reduced collagen cross-linking (both result in osteopenia) and can also affect endothelial function, connecting the loss of bone mass to atherosclerosis. The reduction in collagen cross-linking (due to LOX inhibition) results in lens dislocation and possibly other features, such as the marfanoid habitus seen in some patients. ACh: acetyl choline; AHCY: adenosylhomocysteinase;  AMPAR: α-amino-3-hydroxy-5-methyl-4-isoxazolepropionic acid (AMPA) receptor; aPC: activated protein C; ATIII: antithrombin III; B6: vitamin B6 (pyridoxal phosphate); BHMT: betaine homocysteine S-methyltransferase; CBS: cystathionine ß synthase;  CGL: cystathionine γ-lyase; CH3: methyl group; DNMT: DNA methyltransferase; ET-1: endothelin-1; FV: factor V; FXII: factor XII; Hcy: homocysteine;  HTL: homocysteine thiolactone; IL: interleukin; K160 PTL: posttranslational modification of lysine at position 160 of type 1 collagen; LOX: lysyl oxidase; MAT: methionine adenosyltransferase;  MCP: monocyte chemoattractant protein; Met: methionine; MIF-1:  macrophage migration inhibitory factor-like protein; MS: methionine synthase; mTOR: mechanistic target of rapamycin; NFkB: nuclear factor kappa B; NMDAR: N-methyl-D-aspartate (NMDA) receptor; NO: nitric oxide; OCN: osteocalcin; OPN: osteopontin; PAI: plasminogen activator inhibitor; PKMT: protein lysine methyltransferase; PRMT: protein arginine methyltransferase; ROS: reactive oxygen species; SAH: S-adenosylhomocysteine; SAM: S-adenosylmethionine; TF: tissue factor; tPA: tissue plasminogen activator; THF: tetrahydrofolate; TM: thrombomodulin; UPR: unfolded protein response; VDR: vitamin D receptor; vWF: von Willebrand factor; HCU: homocystinuria; Hcy: homocysteine Image credit: Alkaissi H and McFarlane SI; Created with BioRender.com

Methylation can affect all major players of the "central dogma" of molecular biology, namely DNA, RNA, and protein (Figure 2A). On the DNA level, cytidine methylation at 5C (m5C) by DNA methyltransferase (DNMT1, DNMT3A, and DNMT3B), all of which are SAM-dependent. SAH can inhibit the process of DNA methylation. Thus, the epigenetic processes are altered in HCU with an abnormal SAM/SAH ratio due to higher SAH affinity to DNMT than SAM. Several genes can be epigenetically modified with altered expression in HCU [14]. 

On the RNA level, exciting new fields are being explored with the discovery of RNA methyltransferase machinery (METTL3 complex), "writers" of RNA epi transcriptomics, that adds methyl on adenine (m6A), one of the most abundant RNA modifications. Similar to the epigenetic "eraser," an equivalent epi transcriptomic "eraser" has been identified that removes the methyl group, such as FTO and ALKBH5 proteins. Several RNAs can be methylated with this machinery, such as mRNA, tRNA and rRNA, snRNA, and others, all of which are SMA-dependent. Such epitranscriptomic changes can alter RNA stability (more stable vs. decay) and translation (silenced vs. translated). Data on RNA changes in HCU are lacking and can present a fertile area for research [27].

On the protein level, one can view two amino acids in two main protein methylation processes. The two amino acids that are methylated are lysine with over 50 lysine methyltransferase (PKMT) and arginine with at least 11 arginine methyltransferase (PRMT); both are similar to the previously mentioned methyltransferases, are SAM-dependent. Such post-translational modifications are common in histone and non-histone proteins. In histones, the addition of methyl groups can silence gene expression, such as the famous addition of methyl groups on the ninth and 27th lysine residues of H3 histone protein (H3K9me and H3K27me), done by G9a/GLP and EZH2, also known as "writer" proteins respectively, both of which are SAM-dependent. SAH has been recently shown to reduce EZH2 activity leading to reduced H3K27 methylation, a finding seen in proatherogenic conditions, leading to disinhibition of nuclear factor kappa B (NFkB) expression and a net pro-inflammatory effect [14]. 

Non-histone protein methylation is also common. One such example is the effect of SAH-induced hypomethylation of PGC-1α, a central effector of exercise-induced physiological changes with the overall effect of improved insulin sensitivity and white adipose tissue beige (brown), which has desirable metabolic effects. Interestingly, one of the "read-outs" and effectors of PGC-1α signaling is the myokine ß-aminoisobutyric acid (BAIBA) that increases after exercise, which in our patient levels were doubled after treatment, and reduction of Hcy levels [28,29]. One possible explanation is that the treatment normalized the one-carbon metabolism and alleviated the methyl-donor deficit state, leading to the normalization of PGC-1α signaling and increased BAIBA levels. 

With an advancing field of systems biology, bioinformatics identifies "chromatin organization" as the most affected pathway in Hcy and homocysteine thiolactone (HTL)-treated cell lines data. Affected genes are predominantly involved in cardiovascular biology, explaining the end organ target in HCU. A comprehensive review by Kaján and Jakubowski goes over the accumulating evidence of epigenetic changes being at the core of hHcy and its pathology [30].

Lee et al. reported contradicting results in their study of a transgenic mouse model of zinc-inducible *CBS*. Using methylated DNA immunoprecipitation (MeDIP), they found that DNA methylation levels increased in several genes, contrary to their hypothesis, which suggested that reduced SAM/SAH ratio would inhibit DNA methylation due to SAH's strong binding to DNMT. However, the altered ratio in the transgenic model was accompanied by higher levels of SAM compared to wild-type animals, which may have led to an overall increase in DNA methylation [31].

HTL and Protein Homocysteinylation

Another way in which Hcy exerts its deleterious effect is by forming a highly reactive ringed structure called HTL, which can incorporate into proteins by a dose-dependent, non-enzymatic posttranslational medication process known as N-homocysteinylation, where HTL binds to lysine residues of proteins (Figure 2A) [32,33]. Protein N-homocysteinylation leads to loss of protein function and, eventually, multimerization and precipitation of proteins. Some proteins are more susceptible than others; for example, homocysteinylation of only 33% of lysine residues of methionine-tRNA synthase (MetRS) leads to full inactivation, while other proteins, such as trypsin, are more resistant and would lose function after homocysteinylation of over 80% of their lysine residues [34]. Lysyl oxidase is particularly susceptible to inhibition by N-homocysteinylation, which has implications for vascular and bone damage [35]. Low-density lipoprotein (LDL) is another target of N-homocysteinylation, which induces an immune reaction in animal models of HCU, which may partly contribute to accelerated atherosclerosis. Although certain proteins are less prone to precipitation with homocysteinylation, such as fibrinogen, extreme elevation of HTL will eventually lead to insolubility and precipitation, which may contribute to the thrombophilia seen in HCU patients [34]. Despite the importance of N-homocysteinylation of proteins and its impact on protein function, only recently a robust method of studying protein homocysteinylation was developed, with not much human data on the proteomic level, with *Saccharomyces cerevisiae* being the first organism to have N-homocysteinylation studied on proteome level [36,37].

End-organ damage

Vascular Complications

One of the earliest recognized complications of HCU (Figure 2B). McCully described accelerated atherosclerosis in post-mortem examination of patients with HCU [38]. One of the main aims of lowering Hcy in HCU patients is to prevent recurrent thrombosis, with Hcy target of less than 100 µmol/L. The vascular complications can be divided into two main sections, accelerated atherosclerosis, and thrombophilia. Regarding thrombophilia, hHcy affects two arms of Virchow's triad: endothelial dysfunction and hypercoagulable state.

**Figure 3:**
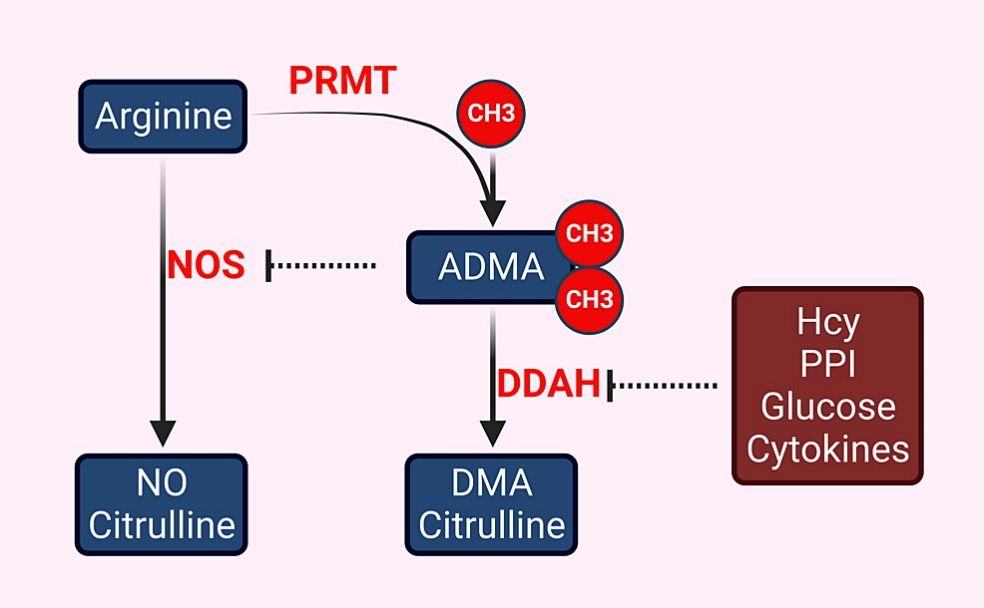
One of the ways Hcy leads to endothelial dysfunction is by reducing NO synthesis. NO is produced from arginine by the action of the enzyme NO synthase (several isoforms exist). A modified arginine, namely dimethylarginine (symmetric and asymmetric), can result from the action of the enzyme PRMT. ADMA can compete with the unmodified arginine on the active site of the NO synthase, thus reducing NO. ADMA is naturally catabolized by the enzyme DDAH to dimethylamine and citrulline. Several DDAH inhibitors result in ADMA accumulation, thus reducing NO. Such inhibitors, such as Hcy, glucose, inflammatory cytokines, and PPIs, may lead to endothelial dysfunction and accelerated atherosclerosis. This might be one of the mechanisms underlying the increase in cardiovascular risk in patients on long-term use of PPI. ADMA: asymmetric dimethylarginine; CH3: methyl group; DDAH: dimethylarginine dimethylaminohydrolase; DMA: dimethylamine; Hcy: homocysteine; NO: nitric oxide; NOS: nitric oxide synthase; PPI: protein pump inhibitors; PRMT: protein arginine methyltransferase Image credit: Alkaissi H  McFarlane SI; Created with BioRender.com

Endothelial dysfunction and accelerated atherosclerosis: Inflammation, endothelial dysfunction, and vascular smooth muscle proliferation lie at the core of atherosclerosis. Endothelial dysfunction in hHcy and HCU is driven mainly by reduced nitric oxide (NO) and increased reactive oxygen species (ROS) production. The enzyme nitric oxide synthase (NOS) derives NO from the amino acid arginine. Arginine can be methylated into symmetric dimethylarginine (SDMA) and asymmetric dimethylarginine (ADMA), which can compete with arginine on the active site of NOS, leading to NOS inhibition and reduction of NO (Figure 3). Normally, ADMA is metabolized by dimethylarginine dimethylaminohydrolase (DDAH), which is inhibited by several molecules known to increase cardiovascular risk, including Hcy. Other famous inhibitors of DDAH include hyperglycemia, inflammatory cytokines, and proton pump inhibitors, leading to a similar state of ADMA accumulation, NOS inhibition, NO reduction, and endothelial dysfunction [39-41].

Hcy inhibits DDAH in a dose-dependent fashion. This inhibition acts on both DNA and protein levels. On the DNA level, Hcy inhibits DDAH at the expression level by methylating the promotor of DDAH. This model has been rescued by the use of methylation inhibitors [42]. On the protein level, Hcy attacks the critical sulfhydryl group in the DDAH enzyme, impairing its action to catabolize ADMA [43]. This model has been rescued by transfecting endothelial cells with an over-expressed DDAH enzyme, leading to the normalization of NO levels in Hcy-challenged endothelial cells [44]. Clinically, higher ADMA levels have been documented in pediatric patients with HCU [45]. However, contradicting evidence regarding ADMA levels in HCU exists, as in one study of 22 patients with HCU, Hcy levels median of 33 (range 14-237 µM) had normal ADMA. One possible explanation for the contradicting data on the Hcy-ADMA correlation is the discrepancy in Hcy levels. The patients with normal ADMA had much lower Hcy levels than untreated hHcy patients, as in cases where the diagnosis is missed [46]. 

This discrepancy might also be attributable to different renal functions, as in one study on individuals with classic HCU secondary to *CBS* mutation, those with impaired renal function, estimated by elevated cystatin C, had higher ADMA as compared to those with normal renal function [47].

Hcy can also reduce the expression of NOS. The effect of Hcy on vascular tone can be further amplified by increased expression of the potent vasoconstrictor endothelin-1 (ET-1). Furthermore, Hcy increases ROS such as superoxide anion (O2•-) and peroxynitrite (ONOO-), leading to increased production of vasoconstrictive eicosanoids such as thromboxane A2 (TxA2). The overall outcome is reduced vasodilators and increased vasoconstrictors [48]. Hcy induces apoptosis in endothelial cells through Fas, p53/Noxa, and cytochrome-c. Additionally, N-homocysteinylation, protein aggregation, misfolding-induced endoplasmic reticulum (ER) stress, and unfolded protein response (UPR) culminate in cell death [49]. The increase in endothelial apoptosis is met with an inefficient endothelial cellular regeneration and growth in hHcy condition, mediated by epigenetic methylation of CpG island in the promotor region of cyclin A gene, resulting in reduced cyclin A [50]. Hcy activates NF-κB with subsequent increases in several cytokines and induction of a pro-inflammatory state [51]. Hcy-treated human monocytes exhibited a dose-dependent increase in TNF-α, IL-6, IL-1ß, IL-8, and IL-12 and a reduction in macrophage inhibitory factor (MIF) [52]. Hcy increases MCP-1 and IL-18 mediated by NFkB, both involved in chemotaxis and an influx of inflammatory cells to atherosclerotic plaques [53]. Further evidence also points toward N-homocysteinylation and the effect of thiolactone on LDL, rendering the molecule more atherogenic [38]. The Hcy-induced NO dysregulation can be reversed by the gasotransmitter H_2_S, produced by the action of CBS and CGL enzymes on Hcy. H_2_S increases NO levels and reduces the permeability of endothelial cells by increasing claudin-5 expression [54].

Lysyl oxidase (LOX) is an enzyme that plays a role in collagen and elastin synthesis and is inhibited by N-homocysteinylation. It has an additional important role in endothelial function and is downregulated by several risk factors such as inflammatory cytokines and hyperglycemia leading to endothelial dysfunction [55]. LOX is also involved in the healing of atherosclerotic lesions [56]. 

On the other hand, betaine treatment may have a beneficial effect in reducing the size of plaques in atherosclerosis mice models. Using double knock-out for apolipoprotein E (apoE) and SAH hydrolase genes, betaine treatment was associated with reduced plaque size and lower levels of CD68+ cells (macrophages) and vascular smooth muscle cells (VSMC) within the plaques. Additionally, the study found that betaine inhibits the phosphorylation of two key signaling pathways, NFkB and ERK, which are known to play a role in inflammation and cell proliferation. Furthermore, the study suggests that SAH-induced endothelial dysfunction may be mediated through the NFkB pathway. Overall, these findings provide evidence that betaine may have therapeutic potential in treating atherosclerosis and suggest that its mechanism of action may involve inhibiting inflammatory signaling pathways [57]. In another study, betaine treatment in apoE knock-out mice significantly reduced plaque size and aortic TNFα levels [58].

Hypercoagulable state: Hcy induces a hypercoagulable state through increased procoagulant factors, reduced anticoagulants, and platelet aggregation. In a longitudinal study following two siblings, levels of thrombomodulin (TM) and von Willebrand factor (vWF) were elevated prior to starting treatment of HCU. After treatment with folate and vitamin B6, levels of TM and vWF normalized [59]. Hcy further increased tissue factors, factor V, and factor XII. On the other side, Hcy reduces the anticoagulant mechanisms by reducing protein C activity, plasminogen activator (tPA), and increased plasminogen activator inhibitor (PAI), leading to an unbalanced coagulation system with increased procoagulant factors and reduced anticoagulants activity [48]. The amino acid lysine plays a crucial role in plasmin activity, as plasmin binds to its target through several lysyl residues. In animal models, fibrinogen lysyl residues are homocysteinylated, and this impairs plasminogen activation by tPA [60]. The importance of lysine in the plasmin-based anticoagulation system has been exploited in several pharmacological lysine analogs that inhibit plasmin activity in patients with bleeding tendencies. Lysine analogs, such as aminocaproic acid and tranexamic acid, are commonly used in clinical practice [61].

Low levels of antithrombin III have been observed in two siblings with HCU over the years. However, the defect seemed to be on the protein synthesis level rather than a diminished activity, as high Hcy levels did not inhibit ATIII activity in vitro [6,62]. Additionally, in vitro data shows that extremely elevated levels of Hcy reduce protein solubility, including fibrinogen, leading to protein aggregation [34].

Bone and Musculoskeletal Complications

The epidemiological evidence tying Hcy and osteoporosis arises from the rare and severe classic monogenic HCU and the more common milder degrees of hHcy. In a population-based study of 11,253 person-years, 191 patients had osteoporotic fractures. Higher natural-log-transformed Hcy levels were associated with a 1.4-fold increase in multivariable-adjusted fracture risk, and Hcy levels in the highest age-specific quartile were associated with a 1.9-fold increase in fracture risk. The risk was similar in the cohorts and men and women, and the link between Hcy levels and fracture risk appeared to be independent of other potential risk factors [63]. In another study, 825 men and 1174 women enrolled in the Framingham study were followed for 12-15 years while Hcy levels were measured in stored blood samples. The average Hcy level was 13.4 µmol/L in men and slightly lower in women at 12.1 µmol/L. Age-adjusted incidence rates of hip fractures per 1000 person-years were found to be dose-dependently associated with Hcy levels. The incidence of hip fractures was 1.96/1000 and 9.4/1000 in men and women, respectively, in the lowest quartile of Hcy levels, increasing to 8.1/1000 and 16.5/1000, respectively, in the highest quartile [64].

While epidemiological studies point toward an association between Hcy and osteoporosis, basic science mechanistic studies further elaborate on the causal nature between Hcy and osteoporosis. Hcy is not merely a passive marker of bone density loss but rather an active player in such a process (Figure 2B) [65]. LOX, an enzyme that catalyzes the reaction of deamination of lysine and hydroxylysine to allysine aldehydes, a crucial step for cross-linking of collagen and elastin that is essential for the tensile strength of both bones and blood vessels [55,66]. LOX has other functions, such as activation of platelet-derived growth factor receptor (PDGFR), inhibition of fibroblast growth factor (FGF)-2, and transforming growth factor beta (TGF-β) [67]. In addition to its role in bone matrix synthesis, LOX plays an important role in osteoblasts differentiation in animal models [68]. On molecular levels, LOX is one of the proteins sensitive to inhibition by HTL and N-homocysteinylation. On tissue levels, such LOX inhibition manifests as pathologies in organs that depend on LOX for extracellular matrix synthesis, namely bones and arteries [32,69]. Other diseases with similar tissue tropism (i.e., vascular and bone pathologic processes) include diabetes mellitus and inflammatory conditions with elevated TNF-α. Diabetes and inflammatory milieu inhibit LOX through miR-203, leading to the growth inhibition of mesenchymal pluripotent stem cells and resulting in osteopenia and vasculopathy. As a result, LOX is an intriguing molecule that intersects bone and vascular biology in several diseases [55,66,67]. In animal models, Hcy impacts collagen through the N-homocysteinylation of the K160 lysine residue at the N-telopeptid HCU end of type 1 collagen. This process inhibits pyridinoline cross-linking of collagen, which helps to explain additional connective tissue manifestations of the disease, such as hyperextensibility, ectopic lentis, and low bone density [35].

Hcy can activate N-methyl-D-aspartate (NMDA) receptor (NMDAR) in the bone, leading to increased intracellular calcium and release of matrix metalloproteases in a calpain-dependent mechanism, accelerating bone matrix degradation. Hcy also increases ROS induced by HTL, leading to the inhibition of osteoblasts and activation of osteoclasts, shifting the balance toward less bone formation and more degradation, with a net effect of bone mineral density loss [70]. 

Elevated levels of Hcy can decrease osteocalcin (OCN) and increase osteopontin (OPN) levels on mRNA levels in cultured osteoblasts [71]. OCN is a bone-derived hormone with several local skeletal and systemic endocrine effects [72]. OPN is a highly acidic and negatively charged secreted phosphoprotein belonging to the SIBLING (short integrin-binding N-glycosylated protein) family. OPN protein binds calcium and acts as a "molecular sponge," inhibiting bone mineralization. Furthermore, OPN tithers down osteoclasts to bone resorption sites. In addition to its role in bone metabolism, OPN has also been linked to arterial calcification, potentially explaining the observed association between "weak bones and hard arteries” [73,74]. Some studies suggest that OPN may act as a reactive response to limit ectopic arterial calcification, suggesting that it may have a universal anti-mineralization effect in both bones and arteries [75].

As previously mentioned, altered one-carbon metabolism and methylation are among the effector mechanisms in HCU. Recent research suggests that bone disease in HCU may be partly attributed to a defect in the methylation of peroxisome proliferator-activated receptor gamma coactivator 1-alpha (PGC-1α). Specifically, a hypomethylated PGC-1α has been shown to interact with vitamin D receptor (VDR), which can affect bone metabolism [76].

In animal models of hHcy induced by a high-methionine diet, bone loss was attributed to a progressive increase in RANKL and reduction in OPG, leading to the net effect of increased osteoclastogenesis. Mechanistically, the oxidative stress from high Hcy and low H_2_S resulted in increased c-JUN/JNK activity, with subsequent DNMT1 over-expression. The latter lead to hypermethylation of CpG island in the OPG promotor region, lower OPG levels and reduced osteoblastogenesis, and a concomitant increase in RANKL expression and subsequent increase in osteoclastogenesis. This model was reversed using the H_2_S-donner sodium hydrosulfide (NaSH) or with JNK siRNA [77]. 

Betaine treatment provides additional supporting evidence of Hcy and bone metabolism. Betaine, which recycles Hcy back to methionine by serving as a methyl donor, has been found to enhance osteoblastogenesis. This effect is mediated by activating RUNX2 and the ERK pathway, leading to increased expression of osteopontin, IGF-1, and RUNX2 [78].

Neurological Complications

As mentioned earlier, HCU was first discovered while investigating etiologies of developmental delay in children, indicating that neurological involvement is one of the most salient features early recognized in children with severe elevation in Hcy (such as classic HCU) [4]. Milder elevation of Hcy levels has a debatable correlation with neurological defects, such as cognitive impairment, Alzheimer's disease, and strokes [79]. Patients with C677T polymorphism in the *MTHFR* gene have a higher incidence of Alzheimer's disease and epilepsy [20]. For every one µmol increase in Hcy, the risk of lacunar infarct increases by a factor of 1.04 (with a 95% confidence interval of 1.01-1.07). High Hcy levels were also linked to poorer cognitive function [80]. HCU patients also exhibit psychiatric manifestations, as some patients may present with obsessive-compulsive behavior [81].

Mechanistically, an increase in Hcy levels has been linked to neuronal damage caused by ROS, reduced cytochrome c oxidase secondary to copper chelation, and an increase in pro-apoptotic signals such as p53, Vax, and caspase-3, ultimately leading to neuronal loss (Figure 2B) [82]. Hcy has also been found to increase glutamatergic transmission, leading to excitotoxicity by activating NMDA and AMPA receptors [83]. Interestingly, Hcy appears to have dual effects on autophagy in different tissues. In neurons, Hcy can lead to the activation of mTORC1 and, subsequently, inhibition of autophagy, resulting in the accumulation of proteins associated with neurodegenerative disorders such as phosphorylated tau and ß-amyloid. This phenotype of Hcy-induced neuronal damage can be reversed and rescued with the mTOR inhibitor rapamycin and activator of autophagy such as TAT-beclin 1 [82]. However, Hcy has the opposite effect on the liver, activating autophagy, resulting in liver damage. This effect was indirectly triggered by the reduction of cystic fibrosis transmembrane conductance regulator (CFTR) expression, which normally inhibits autophagy; thus, CFTR reduction results in the disinhibition of autophagy and liver damage. Hcy-mediated CFTR reduction is a result of DNA methylation [84]. Finally, high levels of methionine have been associated with reduced Na-K ATPase activity, reduced acetylcholine levels, and a reduction in neuronal dendrites [85].

Other Complications

High levels of Hcy interfere with the sulfhydryl cross-linking of proteins, including elastin, explaining the ectopic lentis [6]. In a study of nine individuals with classic HCU, BMI was inversely correlated with Hcy and methionine levels and directly correlated with cysteine levels [86].

Hydrogen sulfide, Hcy metabolism, and aging

As we previously mentioned, patients with HCU exhibit accelerated atherosclerosis, neurodegeneration, and osteoporosis, three pathological processes that represent a hallmark of HCU and aging. As such, HCU can be viewed as a form of "accelerated" aging. HCU pathogenesis is often viewed through the lens of "excess" of Hcy and its downstream effect. However, the pathogenesis of other inborn errors of metabolism also stems from a "deficiency" of downstream metabolic products. In classic HCU, the transsulfuration defect that leads to excess Hcy also results in lower cystathionine and its products, cysteine, taurine, glutathione, and the gasotransmitter H_2_S. 

In 1998, the Nobel Prize in Medicine and Physiology was awarded to Robert Furchgott, Louis Ignarro, and Ferid Murad for discovering NO's role and related signaling in cardiovascular physiology. Not only did this discovery open a wide array of inquiries into NO physiology, but also it was the beginning of a field known as "gasotransmitters" [87]. Hydrogen sulfide is a gasotransmitter; it is the third in this class; its physiologic role was discovered after NO and CO. However, these gasotransmitters (i.e., NO, H_2_S, and CO) were known for their toxic effect long before their role in physiology. These gasotransmitters exhibit a phenomenon where too low or too high concentrations are pathogenic, but at normal levels, they exhibit several physiological benefits. This phenomenon has been known as the "hormesis paradigm” [88].

**Figure 4:**
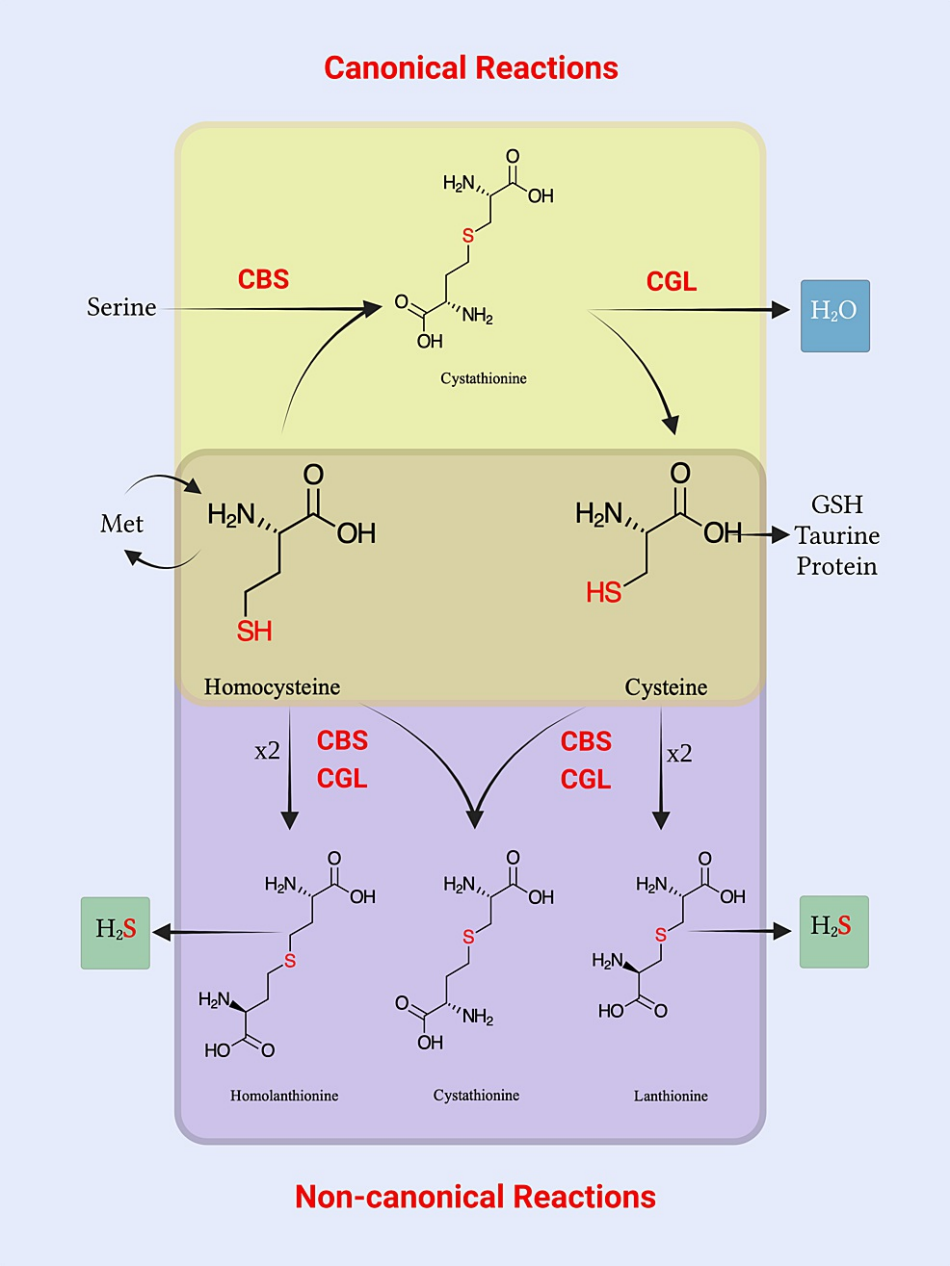
Hcy can either be remethylated to methionine or pass through the transsulfuration pathway to produce other sulfur-containing amino acids (cysteine, glutathione, and taurine.). In these cases, the sequential action of the CBS enzyme on Hcy, then the CGL enzyme on cystathionine, is described here as a “canonical” reaction. The byproduct of these reactions is water. Alternatively, CBS and CGL enzymes can engage in “non-canonical” reactions, where they would condense two Hcy molecules to produce homolanthionine or condense two cysteine molecules to produce lanthionine. CBS and CGL enzymes can also condense cysteine and Hcy (instead of serine), producing cystathionine. All these non-canonical reactions produce H2S as a byproduct instead of water, and they are some of the main pathways to produce the gasotransmitter H2S in humans. CBS: cystathionine ß synthase; CGL: cystathionine γ-lyase; H_2_S: hydrogen sulfide; GSH: glutathione (reduced); Met: methionine; Hcy: homocysteine Image credit: Alkaissi H and McFarlane SI; Created with BioRender.com

H_2_S is derived from enzymes of the transsulfuration pathway (Figure 4). The cytoplasmic enzymes CBS and CGL have other non-canonical reactions, where they promiscuously condense sulfur-containing amino acids such as two cysteines forming lanthionine, two Hcys forming homolanthionine or cysteine and Hcy forming cystathionine (Figure 2). H_2_S is produced in each of these condensation reactions. The serine:cysteine ratio is the major determinant of which reaction CBS would catalyze (canonic vs. non-canonic) [89]. A mitochondrial enzyme, 3-mercapto pyruvate sulfurtransferase, plays an additional role in H_2_S production [90]. 

Physiologically, hydrogen sulfide plays an important role in salvaging oxidized proteins (post-translational modifications induced by ROS). The thiol group of proteins cysteine amino acid undergoes oxidation into sulfenic acid (-SOH), which can be reversed into an inert harmless persulfide (-SSH) with the help of H_2_S in a protein per sulfidation process. However, if sulfenic acid (-SOH) is not reversed, it will form -SO2H and -SO3H, which are irreversible deleterious protein modifications.

A recent work investigating the mechanism behind the anti-aging agent rapamycin in nematodes suggested a novel pathway other than the known mechanistic target of rapamycin (mTOR) inhibition and reduced protein synthesis. Treatment with mTOR inhibitors resulted in the upregulation of transsulfuration enzymes, predominantly CGL, with elevation in H_2_S levels. This effect was mediated by ATF-4 protein expression, and the longevity effect was nulled by CGL loss of function [91]. The ATF4-CGL-H_2_S-complex IV-AMPK axis has also been studied in human umbilical vein endothelial cells. Vascular endothelial growth factor (VEGF)-induced angiogenesis was known as a downstream effect of hypoxia, but sulfur-containing amino acid restriction converges into the same pathway [92]. 

H_2_S has been connected to CVDs, osteoporotic bone diseases, neurodegenerative diseases, and aging. Endothelial dysfunction lies at the core of hHcy pathogenesis. This is partly due to reduced NO signaling and increased cellular permeability by reducing claudin-5 expression when human umbilical vein endothelial cells are exposed to high Hcy levels in vitro. H_2_S, produced by the enzymes CBS and CGL, reverse the endothelial damage by increasing NO and claudin-5 [54]. H_2_S in CBS knock-out animal model helps to reverse Hcy-induced cardiac remodeling. These include c-Fos and c-Jun, reducing cardiac fibrosis (TGF-ß and MMP) and cardiac hypertrophy (ß-myosin heavy chain) with improved cardiac hemodynamics [93]. 

Neurologically, defective H_2_S per sulfidation is implicated in several neurodegenerative diseases such as Alzheimer's disease, Huntington's disease, and Parkinson's disease. H_2_S helps degrade misfolded proteins, and the downregulation of H_2_S in Parkinson's disease may underlie the accumulation of parkin proteins [94]. While aging may aggravate ROS-mediated protein modification and aggregation, H_2_S can salvage these processes at earlier stages. H_2_S is reduced in aging brains through the downregulation of the CGL enzyme. H_2_S may counteract several age-related processes [95].

Regarding bone disease, H_2_S has a protective effect and induces osteoblast differentiation. Osteoblasts deficiency in H_2_S results in reduced calcium influx and reduced PKC-ERK-Wnt-ß catenin signaling, thus impairing the differentiation of bone marrow mesenchymal stem cells to osteoblasts [96]. H_2_S donner NaSH mitigates the hHcy-induced osteoporotic phenotype by restoring the balance between RANKL-OPG, thus reducing osteoclastogenesis (epigenetic level) [77].

One interesting idea regarding prior interventions to lower Hcy might be related to the fact that none of these interventions (folate, B12) would increase H_2_S. In fact, vitamin B12 is a natural chelator of H_2_S.

Although one may expect H_2_S to be low in HCU patients, in a few small studies, blood levels of H_2_S were within normal. Possibly due to the activity of the redundant CGL enzyme; yet this does not exclude low H_2_S activity in the tissue of end-organs. Measuring H_2_S and other sulfur-containing metabolites was not possible in stored patient samples, so a prospective study of 10 patients with transsulfuration defect HCU (CBS mutation) and six patients with remethylation defect (cbl-G and cbl-E mutations). The thioester's homolathionine levels were elevated, while lanthionine levels were reduced in CBS defect (CBSD) but unchanged in remethylation defect (RMD). As expected, cystathionine was low in CBSD and elevated in RMD. H_2_S, on the other hand, was only mildly abnormal in the serum and urine of RMD patients while grossly normal in CBSD. While this may not reflect the H_2_S activity in tissue, it may point toward other pathways of H_2_S production in the body, supported by the relaxed nature of CBS and CGL enzymes that use cysteine and Hcy to make H2S. Other known pathways are through the enzyme methanethiol oxidase (MTO) and the mitochondrial enzyme 3-mercaptopyruvate sulfurtransferase [97]. Similar findings were seen in cultured fibroblasts of patients with HCU (CBS defect) [98]. In the CBS knock-out mice model, H_2_S levels were unaltered, likely secondary to the redundant role of CGL in producing H_2_S [99].

High levels of H_2_S result in several toxicities, partly stemming from mitochondrial complex iV inhibition. A similar phenomenon occurs in Down syndrome. While in classical HCU, the mutated *CBS* may result in defective transsulfuration, the extra chromosome 21 in Down syndrome results in a higher dose of expression of *CBS* (as the gene lies on chromosome 21), and hence enhanced transsulfuration. This results in much higher levels of H_2_S, which can be toxic by reversibly inhibiting mitochondrial complex VI and impairing cellular bioenergetics [100]. The enhanced transsulfuration results in functional folate deficiency, manifesting in red blood cells' higher mean corpuscular volume and sensitivity to the methotrexate effect. Like HCU, the sulfur-containing amino acid defect was coupled to defective one-carbon metabolism and DNA methylation. Higher DNA methylation is seen in Down syndrome [101].

In summary, whether H_2_S levels play a role in hHcy-mediated accelerated aging phenotype (cardiovascular disease, bone disease, and neurodegenerative diseases) is a fertile area of research, with much to be determined and explored.

Treatment

In 2016, a group of experts in HCU authored guidelines regarding the general management of patients with HCU. Since up to half of the patients with classic HCU have a pyridoxine-responsive CBS mutation, the first line of treatment is vitamin B6 at a dose of 10 mg/kg/day. After two weeks, Hcy levels are measured again to determine responsiveness. A fall of Hcy levels to less than 50 µmol/L indicates a "full responder" to vitamin B6. Levels higher than 50 µmol/L indicate partial to non-responders and may require other lines of treatment, such as betaine, in addition to methionine dietary restriction. The goal target level of Hcy is to be less than 100 µmol/L for better outcomes and to ensure the reduction of thrombophilia [102]. Although effective in reducing Hcy levels by remethylating Hcy to methionine, the main side effect seen in betaine use is an increase in methionine, which at higher levels (> 1000 µmol/L) can lead to cerebral edema [103].

Future directions and clinical trials

Several lines of treatment are being explored and investigated to increase the mutated CBS enzyme's residual activity or to replace the enzyme and mitigate end-organ damage.

Stabilizing Residual CBS Enzymatic Activity

Various measures can be taken to facilitate the proper folding of mutated CBS enzyme, including the use of osmolytes, altering the porphyrin ring, or inhibiting the proteasome to prevent degradation of the misfolded enzyme, which may have residual enzymatic activity. 

Enhancing HTLDegradation 

Protein homocysteinylation can be mitigated by increasing the rate of HTL degradation, which is naturally achieved by the action of several enzymes. Some HTL-catabolizing enzymes, like paraoxonase 1 (PON1) and homocysteine thiolactonase (HTase), have extracellular activity. In contrast, others have intracellular activity, like bleomycin hydrolase (BHase) and biphenyl hydrolase-like protein (BPHL) [104]. 

HTL Adducts

In addition to its role as a cofactor of the enzyme CBS, pyridoxal-5-phosphate (vitamin B6) can also form an adduct with HTL, thus preventing protein homocysteinylation by HTL. This may directly serve as a disease-modifying agent even in nonresponding patients (i.e., B6 does not reduce Hcy level to less than 50 mmol/L). 

Other Hcy Lowering Agents

N-acetylcysteine (NAC) treatment has been shown to reduce Hcy levels in lead-exposed patients [105]. NAC may also benefit Alzheimer's disease because it increases the glutathione pool, reduces Hcy, and acts as an antioxidant [83]. 

Taurine Supplementation

One of the cysteine products downstream in the transsulfuration pathway is the amino acid taurine, a non-proteinogenic, sulfur-containing amino acid. Taurine supplementation improved flow-mediated dilation (FMD) in HCU patients, especially in patients with a lower FMD baseline of <10% and high Hcy levels of > 125 µM [106]. 

Enzyme Replacement Therapy

Enzyme replacement therapy is another promising avenue in the treatment of HCU. Several preclinical and early clinical trials of CBS and CGL replacement therapies are underway. Future directions for treatment of HCU include gene replacement therapy using various forms such as adeno-associated virus (AAV)-borne or naked cDNA of the human *CBS* gene [107]. Non-human enzymes such as bacterial (*Pseudomonas putida*) L-methionase, also known as methionine γ-lyase (MGL), which humans lack and have been shown to exhibit anti-tumor effect by starving tumor cells of the essential amino acid methionine, and its subsequent role in one-carbon metabolism and genetic methylation [23]. Such treatment may hold promise in the reduction of methionine in HCU patients, the main source of HCy [107].

## Conclusions

hHcy, in its milder form or more severe monogenic form (i.e., Hcy), provides an insight into cardiovascular, bone, and neurodegenerative diseases, three hallmarks of aging. Given the profound end-organ damage, early recognition and treatment of the severe form (Hcy) is paramount. As for the milder forms of hHcy, insight into the epigenetic changes that result from the defective one-carbon metabolism in hHcy may provide insight into why trials aiming at Hcy-lowering have failed to address the clinical need, as such epigenetic changes may serve as long-lasting “memory” in each end organ (bone, arteries, brain). Further research is needed to understand and potentially reverse DNA methylation patterns in hHcy.
